# A longitudinal study of natural antibody development to pneumococcal surface protein A families 1 and 2 in Papua New Guinean Highland children: a cohort study

**DOI:** 10.1186/s41479-016-0014-x

**Published:** 2016-08-15

**Authors:** Jacinta P. Francis, Peter C. Richmond, Audrey Michael, Peter M. Siba, Peter Jacoby, Belinda J. Hales, Wayne R. Thomas, Deborah Lehmann, William S. Pomat, Anita H. J. van den Biggelaar

**Affiliations:** 1 Papua New Guinea Institute of Medical Research, Goroka, EHP Papua New Guinea; 2 School of Paediatrics and Child Health, The University of Western Australia, Perth, WA Australia; 3 Wesfarmers Centre of Vaccines and Infectious Diseases, Telethon Kids Institute, The University of Western Australia, Perth, WA Australia; 4 Centre for Biostatistics, Telethon Kids Institute, The University of Western Australia, Perth, WA Australia; 5 Division of Molecular Biotechnology, Telethon Kids Institute, The University of Western Australia, Perth, WA Australia

**Keywords:** *Streptococcus pneumoniae*, Natural immunity, PspA, Vaccine, Children, Papua New Guinea

## Abstract

**Background:**

Pneumococcal surface protein A (PspA), a conserved virulence factor essential for *Streptococcus pneumoniae* attachment to upper respiratory tract (URT) epithelia, is a potential vaccine candidate for preventing colonisation.

**Methods:**

This cohort study was conducted in the Asaro Valley in the Eastern Highlands Province of Papua New Guinea, of which Goroka town is the provincial capital. The children included in the analysis were participants in a neonatal pneumococcal conjugate vaccine trial (ClinicalTrials.gov NCT00219401) that was conducted between 2005 and 2009. We investigated the development of anti-PspA antibodies in the first 18 months of life relative to URT pneumococcal carriage in Papua New Guinean infants who experience one of the earliest and highest colonisation rates in the world. Blood samples and nasopharyngeal swabs were collected from a cohort of 88 children at ages 3, 9, and 18 months to quantify immunoglobulin G (IgG) levels to PspA families 1 and 2 using an enzyme-linked immunosorbent assay and to determine URT carriage.

**Results:**

Seventy-three per cent (64/88) of infants carried *S. pneumoniae* at age 3 months; 85 % (75/88) at 9 months, and 83 % (73/88) at 18 months. PspA-IgG levels declined between ages 3 and 9 months (*p* < 0.001), then increased between 9 and 18 months (*p* < 0.001). At age 3 months, pneumococcal carriers showed lower PspA1-IgG levels (geometric mean concentration [GMC] 602 arbitrary units [AU]/ml, 95 % confidence interval [CI] 497–728) than non-carriers (GMC 1058 AU/ml [95 % CI 732–1530]; *p* = 0.008), while at 9 months, PspA1- and PspA2-IgG levels were significantly higher in carriers (PspA1: 186 AU/ml, 95 % CI 136–256; PspA2: 284 AU/ml, 95 % CI 192–421) than in non-carriers (PspA1 87 AU/ml, 95 % CI 45–169; PspA2 74 AU/ml, 95 % CI 34–159) (PspA1: *p* = 0.037, PspA2: *p* = 0.003).

**Conclusion:**

Our findings confirm that PspA is immunogenic and indicate that natural anti-PspA immune responses are acquired through exposure and develop with age. PspA may be a useful candidate in an infant pneumococcal vaccine to prevent early URT colonisation.

## Background

Pneumonia is responsible for more than a million deaths in young children every year and most of these deaths occur in the developing world [1, 2]. In Papua New Guinea (PNG), pneumonia is the most common cause of death in children less than 5 years of age [3] and *Streptococcus pneumoniae* is the most common cause of severe pneumonia. Dense upper respiratory tract (URT) pneumococcal carriage in PNG begins within weeks of birth (median age of acquisition of 19 days [4]), is persistent throughout childhood and is associated with increased risk of acquiring pneumococcal diseases [5].

The currently available 10-valent (Synflorix™; GSK Biologicals, Belgium) and 13-valent (Prevenar 13®; Pfizer, USA) pneumococcal conjugate vaccines (PCVs) are effective in reducing URT carriage and preventing invasive disease caused by vaccine serotypes, but result to some extent in replacement carriage with non-vaccine serotypes, which in turn may lead to replacement disease, as was seen with the earlier marketed 7-valent PCV (Prevenar®; Pfizer, USA) [6–10]. In particular, in high-risk areas like PNG where the range of serotypes causing pneumococcal disease has always been broader than in areas of low endemicity, replacement by non-vaccine virulent serotypes is more likely to occur. New generation pneumococcal vaccines offering protection against all invasive pneumococcal serotypes, which could complement PCVs, would therefore be highly advantageous. Several pneumococcal surface proteins with vaccine potential have been identified and are currently the subject of research, including the pneumococcal surface protein A (PspA). PspA is a surface protein that hinders the activation and deposition processes of the host complement system, particularly complement component C3 [11, 12], hence protecting the bacteria from undergoing phagocytosis and clearance [13].

Animal models of carriage and infections have shown that PspA is highly immunogenic and capable of generating protective antibodies against pneumococcal URT carriage and infection [14–17]. The natural development of immunity to PspA in humans has not been extensively studied. Studies in children have been conducted in countries with moderate and low endemicity, including the Philippines, Australia, and Finland: these studies indicated that there is development of serum PspA family-specific immunoglobulin G (IgG) antibodies in association with *S. pneumoniae* exposure through carriage or infection [18–21]. A study by Laine et al. [22] in Kenya, a high-endemicity setting, demonstrated the development of naturally acquired antibodies to PspA in relation to age; however, this study did not look at pneumococcal carriage. In a comprehensive study conducted in mother–children pairs of refugees living on the Thailand–Myanmar border, Turner and colleagues [23] analysed antibody responses to 27 pneumococcal protein antigens, including PspA family 1 and PspA family 2: no associations between pneumococcal carriage and PspA-specific antibodies were found. Compared to the Thailand–Myanmar refugees’ study [23] where the median age of acquiring pneumococcal carriage was 45.5 days, young children in the highlands of PNG are colonised at a median age of 19 days, and all are colonised at least once by the age of 1 month [4]. Age at acquisition may be an important factor determining immune outcomes, considering that in the first few months of life the immune system is undergoing rapid changes: in other words, exposure to bacterial pathogens like *S. pneumoniae* in the first weeks of life may result in a different immunological response than first exposure when a child is a few months old and the immune system has further developed. It remains to be determined whether early and dense pneumococcal colonisation of the URT, as experienced by infants in high endemicity settings like PNG, results in priming of protective immune responses, or alternatively leads to immune tolerance and consequent increased risk of persistent colonisation and disease.

PspA is a conserved protein that is expressed by virtually all *S. pneumoniae* strains; however, the protein shows structural diversity between pneumococcal strains and has been classified into 3 families based on sequence variability of the N-terminal domain of PspA. Although pneumococcal strains expressing family 1 or 2 PspA proteins account for 98 % of clinical isolates, PspA-specific IgG antibodies binding to this highly variable region are family-dependent [24]. We have previously reported that maternal-derived PspA1- but not PspA2-specific antibodies were associated with a higher risk for pneumococcal colonisation in Papua New Guinean infants [4], indicating a possible difference in the frequency of PspA1 *versus* PspA2 expressing pneumococcal strains circulating in this population, or functional differences between these family-specific antibodies.

The aims of this study were to examine the development of naturally acquired IgG antibodies to PspA families 1 and 2 (PspA1 and PspA2) in the first 18 months of life in a cohort of Papua New Guinean infants and to determine the association between early pneumococcal carriage and antibody responses to these proteins. We hypothesise that, due to the high pneumococcal exposure and early URT carriage experienced in our study population, there will be high naturally acquired IgG responses to the PspA proteins at a young age and that these can protect against subsequent carriage.

## Methods

### Study design, setting, and population

This cohort study was conducted in the Asaro Valley in the Eastern Highlands province of PNG, of which Goroka town is the provincial capital. The PNG Institute of Medical Research (PNGIMR) headquarters, laboratories, and small clinic are located next to Goroka General Hospital, the only tertiary hospital in the province.

The children included in the analysis presented here were participants in a neonatal PCV trial that was conducted between 2005 and 2009. A detailed study protocol is published in Phuanukoonnon et al. [25]. In summary, between May 2005 and September 2007 pregnant women were recruited at Goroka General Hospital antenatal clinic and in villages located within an hour’s drive of Goroka town. Inclusion criteria for enrolment of newborns were the intention to remain in the study area for at least 2 years, a birth weight >2000 g, no acute neonatal infection, and no severe congenital abnormality. Children of mothers known to be human immunodeficiency virus positive were excluded. A total of 318 newborns were enrolled, of whom 104 were randomised to receive Prevenar® (PCV7, which includes the serotypes 4, 6B, 9 V, 14, 18C, 19 F, and 23 F) at birth, 1 and 2 months of age (neonatal group); 105 to receive PCV7 at 1, 2, and 3 months of age (the infant group); and 109 were randomised to the control group, not receiving PCV7. All children received their recommended childhood vaccines according to the Papua New Guinean immunisation schedule including Bacillus Calmette-Guérin vaccine at birth; oral polio vaccine at birth, 1, 2, and 3 months; Hepatitis B vaccine at birth, 1, and 3 months; a combined *Haemophilus influenzae* type b, diphtheria, tetanus, whole cell pertussis vaccine at 1, 2, and 3 months; and a measles vaccine at 6 and 9 months. As part of the trial, at the age of 9 months, all children received a dose of the 23-valent pneumococcal polysaccharide vaccine (PPV) (Pneumovax23®; Merck & Co, USA) containing serotypes 1, 2, 3, 4, 5, 6B, 7 F, 8, 9 N, 9 V, 10A, 11A, 12 F, 14, 15B, 17 F, 18C, 19 F, 19A, 20, 22 F, 23 F, and 33. Study infants were followed for 18 months after birth, in which period there were 10 prescheduled trial visits. In addition, village reporters conducted weekly surveillance of study participants in rural areas throughout the first year of life and then fortnightly to age 18 months.

A requirement for children to be included in the current analysis on the development of IgG antibodies to PspA1 and PspA2 in relation to carriage was a complete set of plasma samples and pernasal swabs at 3, 9, and 18 months of age. Due to financial constraints, PspA-specific antibodies were assessed for only 88 children. Selection was based on the sequential ID numbers of children with complete datasets: of these 88 children, 23 children had been randomised to the neonatal PCV7 group, 38 to the infant PCV7 group, and 27 to the control group.

### Bacteriology of pernasal swabs

Standardised methodologies as described previously [26] were used for collection, transportation, and storage of the pernasal swabs and subsequent culturing, identification, and isolation of *S. pneumoniae* [26–28]. Briefly, pernasal swabs were stored in 1 ml of skim milk-glucose-glycerol broth (SMGGB) at -70 °C until further processing at PNGIMR. After thawing and vortexing, 10 μl aliquots of the pernasal swabs in SMGGB were streaked onto horse blood agar, chocolate agar, gentamicin blood agar (5 μg/ml), and bacitracin chocolate agar (300 μg/ml). Plates were incubated overnight (18–24 h) at 37 °C in 5 % CO_2_-enriched atmosphere. Presumptive pneumococcal colonies were then cultured with an optochin disc and confirmed to be *S. pneumoniae* based on their susceptibility.

### Detection and quantification of PspA-specific IgG using an enzyme-linked immunosorbent assay (ELISA)

As described previously [4, 25, 29], venous blood samples were collected in sterile tubes containing 100 International Units of preservative-free heparin to allow the collection and separation of peripheral mononuclear cells and plasma. Samples were spun within 2 h of collection for 10 min at 700 × g. Plasma samples were aliquoted and stored at -20 °C until they were analysed.

PspA1 (family 1, clade 2) antigen was derived from the recombinant Rx1_AA1.0.302_ protein, and PspA2 (family 2, clade 3) from PspA/V-24_AA1.0.410_. A detailed description on the methodology for the expression and purification of these recombinant antigens is described in Francis et al. [4].

Minor changes were made to a previously established ELISA for the detection and quantification of PspA-specific IgG plasma antibodies [4], including using coating concentrations of 1 μg/ml and 0.5 μg/ml for PspA1 and PspA2, respectively, and using the new human anti-pneumococcal capsule reference standard 007sp (National Institute for Biological Standards and Control [NIBSC], UK). The reference standard was serially diluted (two-fold) using a starting dilution of 1/200 for PspA1 and 1/400 for PspA2. In addition, high and low quality control plasma standards (taken from PNGIMR laboratory volunteers) were diluted at single dilutions of 1/400 and 1/1600, respectively, for both PspA antigens, and test samples were serially diluted (two-fold) using a starting dilution of 1/200.

### Statistical analyses

Since PspA1- and PspA2-specific IgG antibody levels were not normally distributed, analyses were performed on log-transformed data. Paired t-tests were used to investigate the changes in PspA1 and PspA2 antibody concentrations between time points. Differences in geometric mean concentrations (GMC) of PspA1- and PspA2-specific IgG between pneumococcal carriers and non-carriers at each time point were examined using independent samples t-tests with the unequal variance assumption. To confirm these differences after accounting for potential confounding by the vaccine group, linear regression models were run with log concentration as the dependent variable and vaccine group membership was included as a covariate. Microsoft Excel version 2010 (Microsoft Corporation, USA) was used for data management and generation of graphical presentations. Statistical analyses were done using the statistical software package Stata 11 (StataCorp LP, USA). Antibody titres are expressed in arbitrary ELISA units (AU/ml).

## Results

### PspA1- and PspA2-specific antibody responses in relation to age

**Fig. 1:**
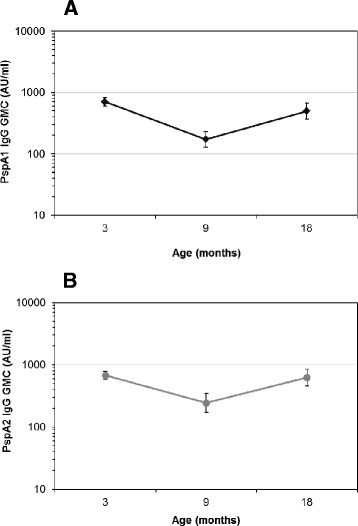
Geometric mean concentrations (GMC) of IgG responses to PspA1 (**a**) and PspA2 (**b**) in relation to age. Error bars indicate 95 % confidence intervals of the GMC. PspA, pneumococcal surface protein A; AU/ml, arbitrary ELISA units/ml

Of the 88 children included in this analysis, 53 were males and 35 were females; 23 were randomised to receive the neonatal PCV7 schedule, 38 received the infant PCV7 schedule, and 27 received no PCV7. As illustrated in Fig. 1a and b, PspA1- and PspA2-specific IgG responses decreased between 3 and 9 months of age (PspA1, *p* < 0.001; PspA2, *p* < 0.001) and then increased between 9 and 18 months of age (PspA1, *p* < 0.001; PspA2, *p* < 0.001).

### PspA1- and PspA2-specific antibody responses in relation to age and URT carriage

**Fig. 2:**
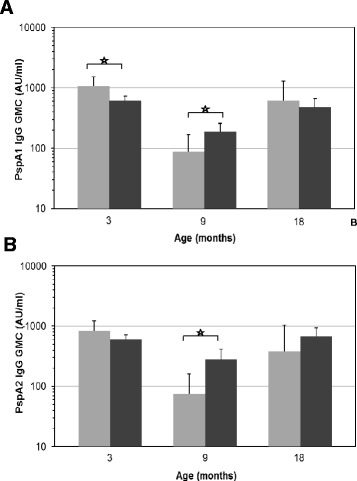
Geometric mean concentrations (GMC) of IgG responses to PspA1 (**a**) and PspA2 (**b**) in non-carrier (*grey bars*) and *Streptococcus pneumoniae* carrier (*black bars*) infants at 3, 9, and 18 months of age. Error bars indicate 95 % confidence intervals of the GMC. ✩ *p* ≤ 0.05 is considered a statistically significant difference. PspA, pneumococcal surface protein A; AU/ml, arbitrary ELISA units/ml

Pneumococcal carriage rates were 73, 85, and 83 % at 3, 9, and 18 months of age, respectively. All children carried *S. pneumoniae* at least once by the age of 18 months. PspA family-specific IgG levels were compared between pneumococcal carriers and non-carriers at the corresponding time points. As illustrated in Fig. 2a, at 3 months of age significantly lower PspA1-IgG levels were found in the pneumococcal carrier group (GMC 602 AU/ml, 95 % confidence interval [CI] 497–728) than in the non-carrier group (GMC 1058 AU/ml [95 % CI 732–1530]; *p* = 0.008). For PspA2-specific IgG responses (Fig. 2b), differences were not significant at age 3 months although responses tended to be higher in non-carriers (GMC 833 AU/ml, 95 % CI 565–1229) than in pneumococcal carriers (GMC 606 AU/ml [95 % CI 509–721]; *p* = 0.127). Conversely, at 9 months of age significantly higher PspA1-IgG responses were found in pneumococcal carriers (GMC 186 AU/ml, 95 % CI 136–256) than in non-carriers (GMC 87 AU/ml [95 % CI 45–169]; *p* = 0.037). IgG responses against PspA2 were also significantly higher in pneumococcal carriers (GMC 284 AU/ml, 95 % CI 192–421) than in non-carriers (GMC 74 AU/ml [95 % CI 34–159]; *p* = 0.003) at 9 months. No significant differences in PspA1-specific IgG responses were observed at 18 months of age between children who carried *S. pneumoniae* (GMC 477 AU/ml, 95 % CI 342–664) or those who did not carry (GMC 618 AU/ml [95 % CI 296–1291]; *p* = 0.503); the same was true for PspA2-specific IgG responses in the carriers (GMC 683 AU/ml, 95 % CI 491–949) and non-carriers (GMC 377 AU/ml [95 % CI 137–1041]; *p* = 0.250).

Linear regression models were run for each of the above comparisons with adjustment for vaccine group. Adjusted effect sizes were similar to unadjusted in all cases and significance levels relative to *p* = 0.05 remained unchanged (results not shown).

## Discussion

To our knowledge this study is the first to examine the natural development of circulating PspA family-specific IgG antibodies in association with age and URT pneumococcal carriage in a high-risk infant population. There were high levels of antibodies at the age of 3 months (most likely due to the presence of maternally derived antibodies) that waned by the age of 9 months. Levels then rose between 9 and 18 months of age, most likely as a result of natural pneumococcal exposure and development of the child’s own immunity.

In a longitudinal study in the Philippines, Holmlund et al. [19] examined natural antibody development to the PspA family 1 protein and the effect of pneumococcal carriage on antibody development in infants aged 6 weeks to 10 months. Children were classified as carriers from the time of first acquisition of *S. pneumoniae*. In this moderate-risk setting, maternally derived PspA1 antibody levels were found to decline to a low at age 20 weeks, followed by a modest increase in circulating antibody to PspA1, which tended to be higher in children who were prior pneumococcal carriers. In our study in the high-risk setting of PNG all children are colonised at least once by the age of 1 month, often with multiple pneumococcal serotypes; a prospective analysis as conducted in the Filipino study [19] is therefore not feasible. The higher PspA-IgG levels we found in non-carrier infants compared to the carrier infants at 3 months of age suggest that maternally derived antibodies may be protective and prevent or clear URT carriage. These high anti-PspA IgG titres early in life already show the potential activity of the protein if given in the form of a maternal vaccination. The higher IgG responses to both PspA1 and PspA2 in the pneumococcal carriers compared to non-carriers at 9 months of age (when maternal antibody has decayed) suggest that infants at this age can generate a good immune response to high levels of pneumococcal exposure. The high antibody titres seen in the pneumococcal carriers in this study cohort at 9 months old may indicate immune priming, but by age 18 months there were no significant differences in antibody titres against both PspA families between pneumococcal carriers and non-carriers. The universal acquisition of the pneumococcus by all Papua New Guinean infants by the age of 18 months is likely to have stimulated the immune response in all children, including the 17 % in whom the pneumococcus was not detected at age 18 months. We suggest that the observed association with pneumococcal carriage as well as the anticipated difference in immune maturation between individuals at 9 months of age can explain the larger variation in PspA-IgG concentrations that we found at this age as compared to when children were younger and responses were dominated by maternal antibodies and when older when their history of exposure and state of immune maturation may be less variable.

Papua New Guinean highland infants continue to experience a high prevalence of carriage, with 73 % of infants already carrying *S. pneumoniae* in their noses by 3 months of age. While we looked at carriage status and PspA antibody response levels in relation to vaccination (because two-thirds of the children in the study had received 3 doses of PCV7), no differences were found, supporting earlier reports from PNG [30] and other high-risk populations [31] that conjugate vaccination has little effect on overall pneumococcal carriage in high-risk populations.

This study has its limitations. Firstly, the sample size of this study is relatively small and there were only 3 sampling points, which limits the power of the study. Another limitation is that we did not assess bacterial load by polymerase chain reaction (PCR). Instead of preventing carriage, it is possible that antibodies acquired to adhesins such as PspA reduce the density of pneumococcal carriage and subsequent risk of invasive disease. To date, financial constraints have precluded (semi) quantitative PCR on any of the nasopharyngeal samples collected as part of our trial. Finally, this study was not designed to determine immune hyporesponsiveness following PPV. However, hyporesponsiveness is unlikely to occur to a protein antigen following PPV vaccination. Despite its limitations, this study does contribute to the existing literature on naturally acquired antibody responses to PspA and their relationship to pneumococcal carriage by providing novel data for children who, with a median age of acquisition of 19 days, represent a population with the earliest documented onset of pneumococcal carriage. Yet, additional studies examining the relationship between development of PspA antibody responses, pneumococcal carriage (including load) and disease are needed in low- and high-risk populations to better understand the immune potential of this virulence antigen.

## Conclusion

High and persistent URT *S. pneumoniae* carriage remains a burden and predisposing factor for early acquisition of pneumococcal diseases in Papua New Guinean children. The development of naturally acquired IgG antibodies against PspA increases with age as a result of active URT carriage exposure. The protective role of naturally acquired immunity to PspA against pneumococcal disease is not clear. Administering a pneumococcal protein-based vaccine containing multiple pneumococcal proteins including potentially PspA1/2 as a complementary vaccine to currently available PCV to offer universal serotype-independent protection against early pneumococcal carriage and disease, may improve vaccine-induced prevention of pneumococcal disease in high-risk populations.
